# The Rise of the Xenes: From the Synthesis to the Integration Processes for Electronics and Photonics

**DOI:** 10.3390/ma14154170

**Published:** 2021-07-27

**Authors:** Carlo Grazianetti, Christian Martella

**Affiliations:** CNR-Institute for Microelectronics and Microsystems, Unit of Agrate Brianza, Via C. Olivetti 2, I-20864 Agrate Brianza, Italy

**Keywords:** Xenes, 2D materials, electronics, photonics, processing, SEDNE, UXEDO

## Abstract

The recent outcomes related to the Xenes, the two-dimensional (2D) monoelemental graphene-like materials, in three interdisciplinary fields such as electronics, photonics and processing are here reviewed by focusing on peculiar growth and device integration aspects. In contrast with forerunner 2D materials such as graphene and transition metal dichalcogenides, the Xenes pose new and intriguing challenges for their synthesis and exploitation because of their artificial nature and stabilization issues. This effort is however rewarded by a fascinating and versatile scenario where the manipulation of the matter properties at the atomic scale paves the way to potential applications never reported to date. The current state-of-the-art about electronic integration of the Xenes, their optical and photonics properties, and the developed processing methodologies are summarized, whereas future challenges and critical aspects are tentatively outlined.

## 1. Xenes: Background and State-of-the-Art

The Xenes family, namely those two-dimensional (2D) materials akin to graphene, quickly expanded in the last 10 years and since the discovery of silicene in 2012 [1] and a new flourishing field of condensed matter physics and materials engineering took off [2]. If it is well-known that carbon can arrange as graphene sheet or diamond depending on the hybridization of the *s* and *p* orbitals, it is not so trivial to figure out a honeycomb lattice for elements such as silicon, germanium or tin. Moreover, it is even more surprising to think about such a crystal structure for elements out of the IVA column (group 14) of the periodic table. However, all of this happened and probably the best is yet to come. Here, the main properties of the Xenes family are reviewed and potential outlook on their applications in nanotechnology is outlined. Before the booming interest generated by the seminal paper on the graphene exfoliation from bulk graphite [3], a theoretical work investigated the possibility to arrange silicon and germanium in a honeycomb lattice [4]. Even though the increased atomic mass with respect to that of carbon, the answer is that, including a buckling in the honeycomb lattice, both silicon and germanium in a graphene-like fashion are possible [5]. More in details the degree of buckling can be tuned, and then low-buckled and high-buckled configurations occur with the former more stable than the latter, of course, low and high are roughly terms for vertical displacements precisely calculated in the specific cases. For freestanding silicene (germanene) low and high buckled lattices show a vertical displacement of 0.44 (0.64) and 2.13 (2.23) Å, respectively [5].

**Figure 1:**
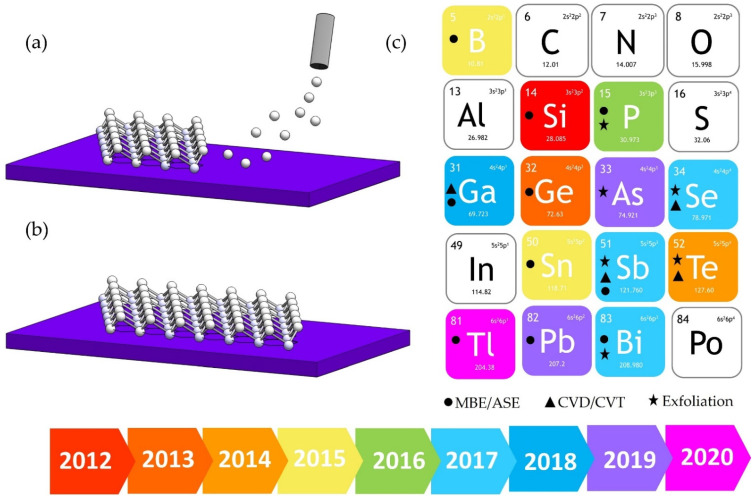
Schematics of the Xenes growth by epitaxy onto a commensurate substrate and Xenes periodic table. (**a**) The X element (white spheres) is typically evaporated on the supporting substrate whose symmetry imposes the buckled honeycomb structure to the incoming atoms provided that the substrate temperature is correctly set. (**b**) The buckled honeycomb lattice appears as a surface reconstruction of the underlying substrate. (**c**) The Xenes periodic table reporting the Xenes discovered so far (from 2012 to 2020) and the growth methods used for their synthesis. In column IIIA there are: borophene, gallenene and thallenene. All the elements below carbon in column IVA have a 2D counterpart. Phosphorene, arsenene, antimonene and bismuthene are the pnictogens Xenes (column VA). In column VIA selenene and tellurene are the chalcogens Xenes. The growth methods (MBE/ASE, CVD/CVT or exfoliation) for every Xene are also reported. It should be mentioned here that we do not have the intention to provide a complete list as the field is evolving rapidly. At the bottom, the timeline evolution of the Xenes discoveries from 2012 to 2020.

Even if freestanding silicene and germanene can exist, there are not bulk silicon and germanium crystals made of silicene and germanene slices to be peeled off. An alternative synthesis method is necessary. The physical vapor deposition (PVD) methods contributed to the realization of the most important advancements in the electronics and photonics fields. High-quality epitaxial layers allowed to the ultrascaling tasks increasing the performance of the devices [6]. In this framework, the improved skills in the deposition of ultra-thin films on appropriate substrates turned out to be the successful key to synthesize Xenes. Noble metals (111)—terminated surfaces for symmetry and chemical reasons were, and still are, the main candidates to the purpose. In particular, to our knowledge, the Ag (111) surface is today the template that can host the large number of Xenes (see schematics of the Xenes deposition in Figure 1). Using molecular beam epitaxy (MBE), silicene [1], germanene [more precisely by atomic segregation epitaxy (ASE)] [7], stanene [8], borophene [9,10], antimonene [11] were successfully realized. Notably, in the case of silicene (but extension to other Xenes is quite straightforward), MBE proved to be effective in the growth of multi-layer silicene, i.e., the multiple integer deposition of single-layer, thus allowing to finely tune the number of layers, even if the precise vertical stacking and its crystal structure are still debated in literature [12,13]. On the other hand, it is intriguing that many of these Xenes were also disclosed on other substrates thus making their existence not linked to a specific template and moreover with other growth techniques out of MBE such as chemical vapor deposition/transport (CVD/T) or even exfoliation (Figure 1c). Silicene was proved to host Dirac fermions when grown onto an insulating substrate such as Al_2_O_3_(0001) [14], stanene was deposited onto the topological insulator Bi_2_Te_3_ [15], Cu(111) [16] or Al_2_O_3_(0001) as well [17], and finally antimonene was realized also on conventional semiconductors such as Ge(111) with different growth techniques [18,19]. The choice of the substrates is of paramount importance as it can determine the properties of the deposited Xene, especially via chemical interaction between the X atoms and the substrate. For instance, stanene on Bi_2_Te_3_ is metal in character whereas when grown on Cu(111) its topological properties are disclosed due to its ultra-flat configuration [15,16]. In this scenario, the buckling is an additional parameter to tune the properties of these new 2D materials. Moreover, the wealth of the electronic states that can be found among the Xenes makes them particularly appealing for device applications (see below). Borophene and gallenene are metal [9,10,20], from silicene to stanene/plumbene the increasing atomic mass of the X element turns the Dirac semimetal into quantum spin Hall insulators such as bismuthene [21,22], and finally phosphorene, arsenene, antimonene, selenene and tellurene are the Xenes candidates in the harsh challenge of 2D semiconductors competing with transition metal dichalcogenides (TMDs) [23]. The Xenes portfolio then includes different electronic properties and offers the major advantage of monoelemental crystals that, on the one hand, makes easier their characterization independently of the environment they are grown and, on the other hand, provides the intriguing opportunity of assembling new materials in the fashion of heterostructures such as the recently reported silicene-stanene [24]. In this scenario, the following challenges are expected to involve the research groups working on the Xenes. First, despite many examples of Xenes grown on different substrates and with different growth techniques, their deposition on large scale substrates (ideally the wafer scale compatible with the semiconductors industry) with easier and cheaper deposition tools is highly demanded. This is an important step towards the integration of these new materials into existing technologies, as already proposed for graphene [25]. Second, many Xenes mentioned before are not stable (mostly in ambient conditions). Improving their stability is therefore of fundamental importance for their integration into devices and this issue can be carried out by different substrates whose interaction with the specific Xene is able to passivate its chemical bonds [2] or by engineering device-ready configuration in which the Xene is appropriately sandwiched in between functional layers either on top or at the bottom [26]. Third, the technology readiness level of the devices based on Xenes should be raised in a short time to prove their competitiveness with the broad library of 2D materials. In the subsequent sections, we will briefly review the recent findings on the Xenes and we will outline their potential applications.

## 2. Xenes for Electronics

The new discoveries about Xenes find a quite natural application in the field of electronics, in particular for silicene that might represent a new life for silicon in nanotechnology [27]. Indeed, the technological urgency to progressively shrink devices to the atomic scale following the recommendations of the so-called Moore’s law questioned the further scalability of the SiO_2_/Si interface, probably the most studied interface of the electronic era since the fabrication of the field effect transistors (FETs) [28]. In this framework, the rise of graphene and 2D materials has been considered the most valuable option to the mentioned issue. Since both the thickness and roughness of 2D layered materials can be controlled accurately at the atomic scale, the reliability and uniformity of the electronic devices based on such materials and their heterostructures could also be optimized [29]. Since the integration of the two most representative 2D materials, i.e., graphene and single-layer MoS_2_ [30,31], a plethora of studies have been reporting the fabrication of 2D materials-based electronic FETs devices with excellent performance that exhibit ON/OFF current ratios >10^7^ and subthreshold swing ~62 mV/decade (or even subthermoionic in tunnel FET [32]) [33]. 

**Figure 2:**
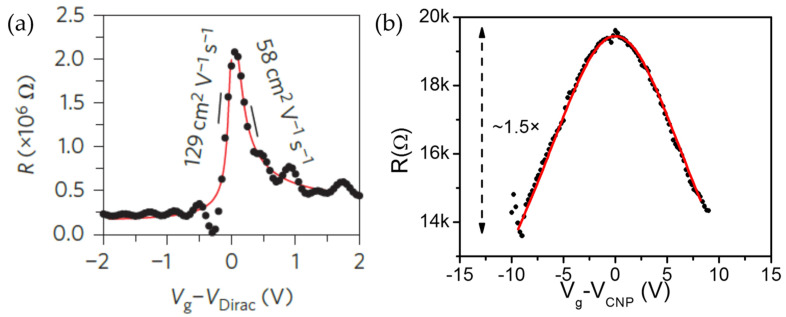
Resistance versus voltage overdrive measured transfer characteristics of single- and multi-layer silicene FETs. (**a**) By means of ambipolar diffusive transport model typically used for graphene FETs, extracted low-field hole and electron carrier mobilities of 129 and 99 cm^2^·V^−1^·s^−1^ in single-layer silicene device. Adapted with permission from Ref. [34]. (**b**) As for (**a**), 66–200 cm^2^·V^−1^·s^−1^ for 24 monolayers thick FET. Adapted with permission from Ref. [35].

Therefore, the rise of 2D materials used as channel in FETs becomes crucial for future applications, in particular for the ongoing comparison with the already existing technologies such as ultrathin body silicon-on-insulator, that in the near future could potentially be replaced or complemented by 2D materials [25]. However, an option still silicon-based would be greatly preferred by the semiconductor manufacturing companies. After the seminal paper on the silicene synthesis [1], a booming interest in silicene led eventually to its integration into a FET just in 2015 through a process called silicene encapsulated delamination with native electrodes (SEDNE) as illustrated in Section 4 [34]. Subsequently, even multi-layer silicene was integrated into a FET [35]. Even if both single- and multi-layer silicene FETs show modest mobility values (up to 200 cm^2^·V^−1^·s^−1^) and low ON/OFF current ratios (up to 10×) as reported in Figure 2a and b for single- and multi-layer silicene FETs, respectively, the main drawback is related to the limited lifetime of the devices (up to 2 days maximum) at ambient condition. Moreover, further improvement is expected either on the transfer process of silicene or on the lithographic step to provide nanometer scale patterning resolution. In Section 1, the absence of a parent crystal from which exfoliate Xene layers was mentioned as the main hurdle to the synthesis of the Xenes and the main difference with graphene and MoS_2_. However, when dealing with the Xanes, i.e., the hydrogen-terminated Xenes such as graphane for graphene [36], exfoliation methods are possible. The hydrogen-terminated germanene, i.e., germanane, was synthesized for the first time from the topochemical deintercalation of CaGe_2_ [37] and the germanane sheets can be mechanically exfoliated as single- and few-layer onto SiO_2_/Si surfaces. In general, the surface termination might be an effective and viable method for tuning material properties, in particular the optical and electronic ones, enhancing the stability of the Xenes. In this way, germanane-based FETs were fabricated by mechanically cleaving flakes of thickness in between 15 and 90 nm that were subsequently transferred on top of a SiO_2_/Si substrate [38]. The devices show transport in both electron and hole doped regimes with ON/OFF current ratio up to 10^5^ and maximum carrier mobility of 150 cm^2^·V^−1^·s^−1^ at 77 K decreasing to 70 cm^2^·V^−1^·s^−1^ at room temperature. FETs were also fabricated using the group 16 elements such as selenium and tellurium. Both these chalcogen elements have the same crystal structure, in which atoms are covalently connected in a spiral chain along one axis thus forming one-dimensional (1D) structures such as nanowires or nanotubes [39]. The calculated electronic bandstructure shows that selenene is a direct bandgap semiconductor [40] and can be synthetized by PVD on Si(111) substrates [41]. After the usual transfer to a SiO_2_/Si substrate, selenene sheets were tested as channel (thickness 16 nm) in a FET fabricated with a reported stability of days in ambient conditions [41]. The selenene-based FETs exhibit a p-type transport behavior mainly attributed to the presence of hydrogen and hydroxyl terminations on the nanosheets surface, with a high ON/OFF current ratio (~10^6^) and hole mobility of the order of 0.26 cm^2^·V^−1^·s^−1^ at 300 K (this value of the hole mobility is lower compared to other 2D semiconductor materials such as tellurene and black phosphorous [42,43]). According to first-principles calculation, the stable form of tellurene exhibits a semiconducting behavior (with a thickness-dependent bandgap from ~0.35 to 1.2 eV) thus making it appealing for electronic applications [44]. For instance, tellurene sheets, obtained by a solution-based growth, have been used as channel in FET devices [43]. Similar as with the selenene-based devices, the tellurene transistors (even in this case the channel thickness is 16 nm) show a p-type transport characteristic and high ON/OFF current ratio (~10^5^) at room temperature with the extracted field-effect carrier mobility is 700 cm^2^·V^−1^·s^−1^ which is comparable or higher than the value reported for transistor based on black phosphorous or other 2D materials [31,45]. The intrinsic crystal anisotropy of tellurene (the same for selenene) is reflected in the anisotropic in-plane electrical transport along different crystal directions at room temperature. The tellurene-based FETs have also demonstrated a good stability when exposed to air for months without any protecting layers. 

Benchmarking 2D materials FETs has been carried out only with TMDs to date [46,47]. Hence, in order to provide useful technological knowledge on the Xenes-based FETs, the four requirements recently proposed by Lanza et al. can be followed even for the Xenes [46]. They can be summarized and extended to the Xenes devices as: (1) the methods used for the synthesis of the Xenes and the fabrication of the devices must be scalable to wafer level (i.e., mechanical exfoliation of bulk crystals is neglected); (2) the morphology and density of non-idealities in the Xenes used (e.g., thickness fluctuations, lattice distortions) will be clearly specified and statistically demonstrated; (3) the size of the Xenes-based FETs will be small enough to be compatible with the integration density requirements of the target technology; (4) information about yield, device-to-device variability, reliability and stability will be provided. Even if this latter step is yet to come, these findings on the Xenes electronic devices are promising and there is plenty of room to improve their fabrication process in the near future. Indeed, for 2D materials (and for the Xenes in particular), the metrics typically considered for evaluate the device performance could be refined with additional growth and process improvements, but conversely for ultrathin silicon films these are fundamentally limited by thickness variation. In this light the future challenge is therefore devoted to the improvement of the silicene FETs performances (mainly stability) and also to extend the device fabrication processes to other Xenes (see Section 4), thus paving the way to the realization of FETs for flexible [48], low-power [49] and even topological [50] electronics based on the Xenes. Further development on the FET architecture is also expected to be beneficial even for field out of electronics such as biology [51], medicine [52] or human-integrated electronics [53].

## 3. Xenes for Photonics

The advent of 2D materials has enabled the exploration of unprecedent aspects in light-matter interaction. Indeed, novel optical responses stem from the intrinsic quantum confinement of the 2D planes. Moreover, intriguing optical functionalities arise from material engineering, exploiting thickness control or morphology manipulation, combined with the realization of 2D heterostructures [54,55,56]. Xenes, as members of the 2D family, are being extensively studied aiming at the development of nanophotonic applications combining the exotic physical properties at the 2D level, from the optical bandgap tunability to Dirac-like electrodynamics, with the electromagnetic spectrum from the ultraviolet (UV) to the terahertz (THz) range.

Ongoing advances in the synthesis and manipulation of Xenes have enabled the optical characterizations of monoelemental 2D materials, whose properties were only theoretically predicted so far. Indeed, the optical properties of Xenes, such as silicene and stanene, were investigated in the theoretical framework recurring to first-principles calculations [57,58,59]. The calculated optical properties of the isolated 2D silicene have revealed peculiar spectral features similar to the graphene case, in terms of absorbance, due to the linear bands and Dirac cones at low optical frequencies, while for higher photon energies interband transitions near critical points have been predicted to dominate the optical response [59]. Paradigmatic cases are the growths of 2D silicon and tin nanosheets on transparent c-Al_2_O_3_, namely (0001)-terminated Al_2_O_3_, by MBE deposition [14,17].

**Figure 3:**
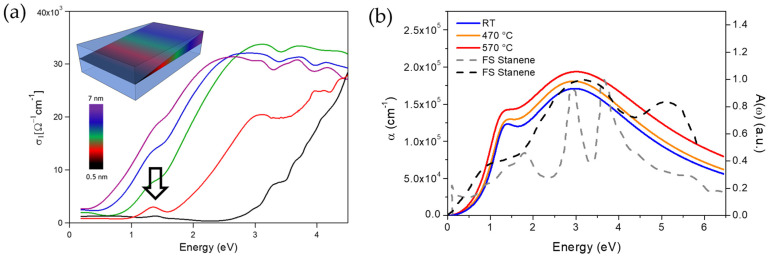
Optical characterization of silicon and tin nanosheets grown on c-Al_2_O_3_. (**a**) real part of the optical conductivity, σ_1_(ω), of silicon nanosheets with different thicknesses (see color scale). Samples with 0.5 nm thickness (black curve) show an absorption peak around 1.4 eV superimposed to a nearly flat background in the whole IR range. Adapted with permission from Ref. [14]. (**b**) Optical absorption coefficients, α(ω), of 0.5 nm-thick tin films deposited at different temperatures, from room temperature to 570 °C. The absorption coefficients show a quite similar shape characterized by two broad peaks centered at nearly 1.25 and 3 eV and are superimposed to two different calculated absorbance spectra of freestanding stanene (gray-dashed from Ref. [57] and black-dashed from Ref. [58]). Adapted with permission from Ref. [17].

Notably, in the infrared (IR) range of the electromagnetic spectrum, the 2D form of silicon showed a Dirac-like absorption resembling the theoretical predicted spectral features of freestanding silicene. In detail, the derived optical conductivity σ_1_(ω) captured the spectral features of interband transitions of the joint density of states, see Figure 3a [14]. In the visible spectral range, σ_1_(ω) drops with a broad gap and rises linearly around 3 eV up to the UV range. The spectral feature around 1.4 eV (empty black arrow in Figure 3a) and the increasing absorption around 4 eV are in good agreement with *ab initio* calculations of freestanding silicene and can be related to two main interband transitions at M (π→π* transition) and at Γ (σ→σ* transition) points of the first Brillouin zone [59]. Increasing the thickness to 1.5 nm (red curve in Figure 3a) leads to a more pronounced peak around 1.4 eV and the onset of absorption at energies around 2 eV. Further increasing the thickness, the low-energy peak progressively disappears but the flat IR background is still visible up to the maximum thickness of 7 nm. Furthermore, the deposited silicon, up to a maximum thickness of 7 nm, showed an anomalous optical behavior characterized by the absence of an optical bandgap at variance with amorphous or crystalline silicon. Similar investigations revealed that the optical response of tin atomically thin nanosheets does not match with that of conventional tin, either in the metallic or oxide fashion, but the absorption spectra showed spectral signatures compatible with calculated spectra of freestanding stanene considering spin-orbit coupling as reported in Figure 3b [17,57,58]. 

These novel insights in the optical properties of Xenes pave the way to their application in nanophotonics with particular interest for the THz spectral range. For instance, the 2D silicon nanosheets on c-Al_2_O_3_ promises to sustain the excitation of plasmonic resonances in the THz range because of the observed Dirac-like electronic character, as previously reported for graphene [60]. At the same time, the ultrathin tin nanosheets are an interesting platform where to exploit the effect of non-trivial topological state in the plasmonic optical response. As a matter of fact, Xenes such as stanene and plumbene, because of their high atomic mass, are expected to host a quantum spin Hall (QSH) insulator state, a 2D topological state where an insulting bulk coexist with conductive edges [21]. Even though initially predicted for graphene, the QSH state has been naturally extended to silicene, germanene, and other Xenes [61,62]. Note that also arsenene, under application of a strain field, is expected to show a QSH insulator state [63]. These results extend the members of the Xene family playing a role in the plasmonic field, which, apart from graphene, include other monoelemental 2D materials endowed with metallic behavior such as borophene, whose plasmons in the IR and visible range have an anisotropic behavior [9,10,64] and gallenene, which showed a tunable plasmonic dispersion as a function of the dielectric environment [20,65].

Combining the variety of optical and electronic properties is the key for the application of Xenes in ultra-scaled photonic devices. For instance, van der Waals epitaxial tellurene on flexible mica showed a good photoresponse in the visible range (wavelength of 473 nm) also after different cycles of substrate bending [66]. Moreover, thanks to the thickness-dependent bandgap (~0.35–1.2 eV), a photoresponsivity of 8–10 AW^−1^ and peak detectivity in the range 1.4–2.4 µm was measured in tellurene nanosheets at room temperature [67]. Selenene phototransistors exhibited good stability in ambient condition and high performances, in terms of spectral sensitivity, detection speed and noise under red laser illumination [41]. In addition to the realization of photoconductive detectors, tellurene and antimonene are used in prototypical biomedical devices for cancer therapy and photoacoustic imaging taking advantage of the strong photon absorption in the visible and near-IR range [68,69]. 

Several Xenes exhibit also relevant non-linear optical properties. Among them, tellurene shows a stark optical Kerr effect [70], while antimonene nanosheets have a giant non-linear refractive index of ≈10^−5^ cm^2^W^−1^ in the visible range [71]. Bismuthene, as well, is extensively studied for the realization of saturable absorber in the technology of ultrafast (femtosecond) IR lasers because of the strong non-linear optical refraction index, which lead to a saturable intensity of 30 mWcm^−2^ [72]. These optical properties make Xenes appealing also for the realization of non-linear photodiode, Q-switching and optical modulators in view of photonic communication system [73]. 

The development of Xene-based opto-electronic devices strictly share the requirements (1)–(4) pointed out in view of the application of Xenes for electronics (discussed in Section 2). Nevertheless, specific technological advances can be specialized in the case of the Xenes photonics. Firstly, the control of light (direction, polarization, phase, etc.) by means of functional devices typically require the definition of complex architecture matching materials with different optical responses, for instance, plasmonic and/or dielectric nanostructures [74]. The integration of Xenes adds an extra-level of complexity in the design step of such a kind of devices because of the intrinsic limitations related to their synthesis and handling [26]. In the next section, a few strategies aiming at addressing these limitations will be presented and discussed. Secondly, and partially overlapped with the challenges described above, there is the need to extend the spectral range of the Xene response thus making possible the integration into state-of-the-art photonic circuit. In this respect, a promising strategy is the realization of vertical and lateral Xene heterostructures [24]. Lastly, similar to TMDs [75,76,77,78], Xenes represent ideal materials where to test the fine-tuning control of the optoelectronic properties by applying external strain or controlled morphological modifications. In this scenario, both experimental and theoretical studies may have dramatic implications in the development of new technological platforms for flexible photonics and quantum communication technologies [74].

## 4. Challenges and Advances in Xene Processing

The development of processing schemes for Xenes technological exploitation poses several challenges. Open questions concern “how” and “to what extent” the available Xenes are compatible with standard device manufacturing. To be realistically suitable for the development of complex device architectures, the control of the Xene quality must be improved in many practical cases. Meeting these requirements could be challenging in exfoliation-based approaches where the thickness and the lateral scale size of the Xene domains are hardly harnessed [20,37,43,79]. Indeed, large-scale deposition techniques should be envisaged and investigated. These alternative approaches are mainly based on chemical or physical vapor methods promising a potential up-scaling of the material deposition on the wafer scale and the translation of the Xene technology from laboratory to fab environment. For example, apart from MBE, antimony nanosheets were realized on Ge(111) by metallo-organic chemical vapor deposition [18], selenene was synthetized by PVD on Si (111) [41], ultra-scaled thin tellurium films were obtained, among the others, by PVD and magnetron sputtering approaches [80,81]. These are preliminary outcomes towards large scale fabrication. 

Other aspect that warrants further investigations in Xene processing is the compatibility with the standard nanofabrication tools. Such an issue includes the establishment of patterning and etching protocols assessing the stability and response of the different Xenes to chemicals and physical agents in use, including high-temperature annealing, dry and wet etching, ion-assisted cleaning and deposition process. As an example, homemade solutions based on potassium iodide/iodine were successfully used for the selective etching of the metal substrate preserving the integrity of the epitaxial silicene layer [34]. However, in many practical cases, the most relevant challenge is to preserve the Xene structural and chemical integrity when brought outside the ultra-high vacuum deposition system. At variance with other 2D materials, such as TMDs and graphene, for which manipulation is relatively easy thanks to a good stability in ambient condition, Xenes require smart solutions enabling protection and portability of the layers. A paradigmatic case, in this respect, is represented by the in-situ encapsulation of silicene by means of an Al_2_O_3_ capping layer [34,82]. The adopted strategy enabled not only to protect silicene in ambient condition, but also the electrostatic gating of Xene-based FET channels (the SEDNE process mentioned in Section 1).

This example clarifies that, beyond a protective layer against Xene degradation, the in-situ deposited capping layer may represent an active player in view of device integration, and not only for silicene [83]. It is therefore reasonable to put forward the idea of a capping layer engineering, exploiting novel materials and deposition methodologies, in view of a target technological application. For instance, high*-k* dielectric materials are important for electronic applications in which electrostatic gating is required. Very recently, the use of a capping layer constituted by insulating 2D material has been proposed as a strategy to implement all-2D based nanoelectronic devices with clean interfaces and improved electrical performances [84]. This strategy may represent a great boon to flexible and wearable electronics [48]. In photonic applications, capping layers with a well-defined optical response must be engineered as a function of the spectral range of interest.

**Figure 4:**
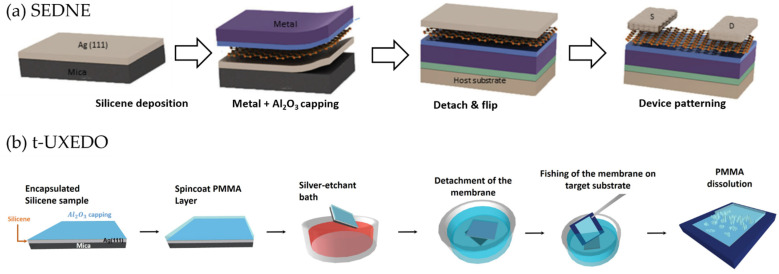
SEDNE and t-UXEDO processes. (**a**) Schematics of the SEDNE and s-SEDNE process developed for the manipulation of a silicene layer grown on Ag(111)/mica substrate. To facilitate the sample handling during the process steps, the robustness of the Al_2_O_3_-encapsulated silicene layer is strengthen via deposition of multilayer stacks made by dielectric (Al_2_O_3_, SiO_2_, etc.) and metal layers. Taking advantage of the mica cleavability, the metal/dielectric/silicene/Ag stack is detached from the substrate and flipped upside down. The pristine silver substrate is then lithographically patterned to create metal contacts aiming at measuring the electrical performances of the silicene-based FETs. Adapted with permission from Ref. [27] (**b**) Schematics of the t-UXEDO process. From left to right: A polymethyl methacrylate (PMMA) polymer layer is spin-coated on top of the encapsulated silicene. The sample is then immersed in a commercial potassium iodide/iodine solution for the etching of the metal substrate. Before the complete etching, the sample is immersed in a de-ionized water bath favoring the mica detachment. The PMMA/Al_2_O_3_/silicene membrane is then transferred onto an arbitrary substrate. The final step is the dissolution of the PMMA layer in acetone or toluene solution. Adapted with permission from Ref. [26].

In view of integration into functional devices, another key milestone is the transfer of the Xene from the native substrate onto arbitrary substrates. As discussed in previous Sections, epitaxy of the Xenes is typically favored by specific crystal orientation of metal surfaces [2]. In this respect, sketches in Figure 4 provide a full picture of the reported methodologies in case of epitaxial Xenes deposited on single crystal (111)—noble metals supported by mica substrates. Even though the process flows include a series of complex steps, it is worth noting that the effectiveness of the approaches stems from two simple experimental strategies. One is the in-situ encapsulation of the Xene discussed above, the other is the cleavability of the mica substrate by means of mechanical and chemical delamination. Both the conditions led to the SEDNE approach (see Figure 4a) first reported for silicene grown on Ag(111) and successfully employed in the realization of FETs operating with both single- and multi-layer silicene channels [34,35]. The SEDNE approach, subsequently revised into the seamless-SEDNE (s-SEDNE), aiming at improving the dielectric gating control, was extended to any epitaxial grown Xenes in the universal Xene encapsulation, decoupling and operation (UXEDO) approach. The latter approach was further combined with the polymer-assisted strategy, developed for TMDs and graphene, into the transfer-UXEDO (t-UXEDO) methodology (reported in Figure 4b), demonstrating for the first time, the transfer of the Xene layer on arbitrary target substrates [26]. Notably, promising advances in the UXEDO methodologies can be represented by the recent reported epitaxial Xene heterostructures [24]. As a matter of fact, the heterostructures not only open the door to the exploitation of multiple vertical stacks of Xenes of different nature, in the so-called “Lego-like” approach [85], but also are a new platform where to test novel approaches for Xene handling. For instance, novel methodologies can be envisaged where one (or more) of the Xene layer forming the heterostructure is considered as sacrificial aiming at improving the reliability of the transfer process.

## 5. Conclusions

The Xenes joined the 2D materials family only recently, but their booming interest supported by preliminary devices demonstration look promising to further explore their exciting properties. Of course, the practice and knowledge on graphene and TMDs made easier to face the Xenes challenge. Indeed, on the one hand, the absence of exfoliation methods to achieve the Xenes motivated scientists to address alternative growth methods in close combination with atomistic and spectroscopic characterization tools. On the other hand, such a survey resulted in a flexible scenario, where the complicated effort to grow the Xenes can be in turn exploited to engineer and manipulate matter. In this framework, the Xenes represent an intriguing opportunity for fields such as electronics and photonics (corroborated by dedicated processing methods) where societal and technological challenges demand for quick, reliable and cheap solutions to everyday life problems in a broad range of human activities including health, energy, communication and so on.

However, major undertakings are still to be addressed before a new era of Xene-based electronics and photonics comes into being, and with them a plethora of exciting opportunities for an interdisciplinary advancement in material science and technology.

## Data Availability

The data presented in this study are available on request from the corresponding author.
